# Carotid Geometry and Wall Shear Stress Independently Predict Increased Wall Thickness—A Longitudinal 3D MRI Study in High-Risk Patients

**DOI:** 10.3389/fcvm.2021.723860

**Published:** 2021-10-26

**Authors:** Christoph Strecker, Axel Joachim Krafft, Lilli Kaufhold, Markus Hüllebrandt, Martin Treppner, Ute Ludwig, Göran Köber, Anja Hennemuth, Jürgen Hennig, Andreas Harloff

**Affiliations:** ^1^Department of Neurology and Neurophysiology, Faculty of Medicine, Medical Center—University of Freiburg, University of Freiburg, Freiburg, Germany; ^2^Department of Radiology—Medical Physics, Faculty of Medicine, Medical Center—University of Freiburg, University of Freiburg, Freiburg, Germany; ^3^Fraunhofer MEVIS, Bremen, Germany; ^4^Institute for Imaging Science and Computational Modeling in Cardiovascular Medicine, Charité-Universitätsmedizin Berlin, Berlin, Germany; ^5^Institute of Medical Biometry and Statistics, Faculty of Medicine and Medical Center, University of Freiburg, Freiburg, Germany

**Keywords:** carotid artery, atherosclerosis, geometry, wall shear stress, magnetic resonance imaging

## Abstract

**Introduction:** Carotid geometry and wall shear stress (WSS) have been proposed as independent risk factors for the progression of carotid atherosclerosis, but this has not yet been demonstrated in larger longitudinal studies. Therefore, we investigated the impact of these biomarkers on carotid wall thickness in patients with high cardiovascular risk.

**Methods:** Ninety-seven consecutive patients with hypertension, at least one additional cardiovascular risk factor and internal carotid artery (ICA) plaques (wall thickness ≥ 1.5 mm and degree of stenosis ≤ 50%) were prospectively included. They underwent high-resolution 3D multi-contrast and 4D flow MRI at 3 Tesla both at baseline and follow-up. Geometry (ICA/common carotid artery (CCA)-diameter ratio, bifurcation angle, tortuosity and wall thickness) and hemodynamics [WSS, oscillatory shear index (OSI)] of both carotid bifurcations were measured at baseline. Their predictive value for changes of wall thickness 12 months later was calculated using linear regression analysis for the entire study cohort (group 1, 97 patients) and after excluding patients with ICA stenosis ≥10% to rule out relevant inward remodeling (group 2, 61 patients).

**Results:** In group 1, only tortuosity at baseline was independently associated with carotid wall thickness at follow-up (regression coefficient = −0.52, *p* < 0.001). However, after excluding patients with ICA stenosis ≥10% in group 2, both ICA/CCA-ratio (0.49, *p* < 0.001), bifurcation angle (0.04, *p* = 0.001), tortuosity (−0.30, *p* = 0.040), and WSS (−0.03, *p* = 0.010) at baseline were independently associated with changes of carotid wall thickness at follow-up.

**Conclusions:** A large ICA bulb and bifurcation angle and low WSS seem to be independent risk factors for the progression of carotid atherosclerosis in the absence of ICA stenosis. By contrast, a high carotid tortuosity seems to be protective both in patients without and with ICA stenosis. These biomarkers may be helpful for the identification of patients who are at particular risk of wall thickness progression and who may benefit from intensified monitoring and treatment.

## Introduction

Preventing the progression and rupture of internal carotid artery (ICA) plaques is of highest importance to avoid ischemic stroke, permanent disability, and death (1, 2). In addition, it has been shown that the progression of the carotid wall thickness over time is associated with an increased risk of ischemic stroke (3). Accordingly, it is of high clinical importance to identify all potential and underlying morphological and hemodynamic parameters, which trigger this cascade independently from established cardiovascular risk factors.

Longitudinal animal studies have convincingly shown that geometry and hemodynamics influence the development of carotid atherosclerosis. Especially, high oscillatory shear stress led to stable while low wall shear stress (WSS) induced rupture-prone “vulnerable plaques” (4). The transfer of such findings to humans is limited because of the use of genetically selected animals, special casts and atherogenic diets. However, they provide important insights into the pathophysiology of atherosclerosis and emphasize the role of such biomarkers.

Cross-sectional studies in healthy volunteers suggested that carotid bifurcation geometry is responsible for the development of atherosclerosis through the formation of “disturbed flow.” Especially a large carotid bulb and a low tortuosity were associated with critical low and oscillating WSS (5, 6). Subsequently, cross-sectional studies in patients (7–9) showed that carotid geometry and hemodynamic factors (10) are independent predictors of increased wall thickness. Moreover, Jiang et al. (11) demonstrated the independent association of carotid geometry with vulnerable plaques representing an imminent risk of brain embolism. However, these studies revealed a partially inverse relationship of geometry in patients compared to healthy volunteers (5, 6) and concluded that geometry plays a role in early stages of atherosclerosis as long as there is no relevant stenosis or inward remodeling (7).

To date, only few longitudinal studies have investigated the influence of carotid geometry and WSS on wall thickness in patients. Cibis et al. (12) studied 14 patients over 4 years using phase-contrast MRI with computational fluid dynamics (CFD) and demonstrated that areas of low WSS were associated with increased carotid wall thickness. Similarly, an ultrasound study showed plaque progression in low WSS areas in 48 patients during a 12-year follow-up (13). Finally, a study using MRI plus CFD in 13 patients undergoing carotid surgery revealed that a large carotid bulb and low WSS were predictors for an increased risk of restenosis during 5 years follow-up (14).

However, a larger longitudinal study in patients demonstrating the independent role of both carotid geometry and WSS on carotid wall thickness is lacking. In addition, previous studies required additional CFD analyses that are based on assumptions regarding vessel compliance and blood viscosity instead of acquiring fully realistic data. Understanding the underlying mechanisms of progression of carotid wall thickness is crucial for the identification, monitoring and optimal medical or surgical treatment of patients with high cardiovascular risk. Thus, we systematically investigated the independent role of geometry and WSS on carotid wall changes in 97 patients with high cardiovascular risk using 3D multi-contrast and 4D flow MRI at baseline and 12 months later.

## Materials and Methods

### Study Population

From April 2018 to February 2019, we prospectively and consecutively screened all patients from our in- and outpatient clinic ≥50 years of age who had hypertension, at least one additional cardiovascular risk factor, and ≥1.5 mm thick plaque of the ICA or distal common carotid artery (CCA) in ultrasound for study inclusion. All patients included in our study had to have a history of hypertension and at least one additional cardiovascular risk factor in order to increase the likelihood of a measurable increase of wall thickness during the 12 months follow-up period.

Exclusion criteria were: contraindications to 3 Tesla MRI such as ferromagnetic implants, claustrophobia, poor clinical condition [modified ranking scale (mRS) > 3 at baseline], atrial fibrillation or other relevant cardiac arrhythmias interfering with the ECG-trigger in MRI, ICA-stenosis >50% (NASCET criteria) (15), life expectancy <2 years, pregnancy, distance to the place of residence >100 km (to reduce the risk of loss to follow-up) and refusal of study participation (9).

We obtained written informed consent from all participants and the local ethics committee approved the study. All ultrasound and MRI procedures were in accordance with institutional guidelines.

### MRI Protocol

Imaging was performed on a 3T whole-body scanner (Prisma, Siemens Healthineers, Erlangen, Germany) using an 8-channel surface coil (NORAS MRI products GmbH, Hoechberg, Germany). MRI examination was performed in all patients with the same scanner and identical protocol at baseline and follow-up. The detailed MRI protocol was described previously (9) and is provided in the supplement.

In brief, the 3D MRI protocol included time of flight (TOF) angiography (spatial resolution 0.5 × 0.5 × 0.6 mm^3^) and T1-, T2-, and proton density (PD)-weighted black-blood imaging (spatial resolution 0.6 mm^3^). For T1-, T2- and PD-weighted imaging a variable-flip-angle 3D Turbo Spin Echo-sequence (Sampling Perfection with Application optimized Contrasts using different flip angle Evolution–SPACE) with fat saturation and dark-blood preparation was used. For blood suppression, a motion-sensitized driven equilibrium (MSDE) preparation was used for T1, T2 and PD imaging (16). 4D flow data (spatial/temporal resolution = 0.8 mm^3^/52.8 ms) was acquired using prospective ECG-triggering and a *k*–*t*-accelerated time-resolved 3D phase contrast sequence (17, 18). The MR-measurement volumes were centered on the flow diverters of both carotid arteries.

We recorded blood pressure levels at the upper right arm of all patients before and after MRI examination after resting in a supine position for at least 5 min and documented heart rate every 4 min during 4D flow MRI.

### Data Analysis

**Figure 1:**
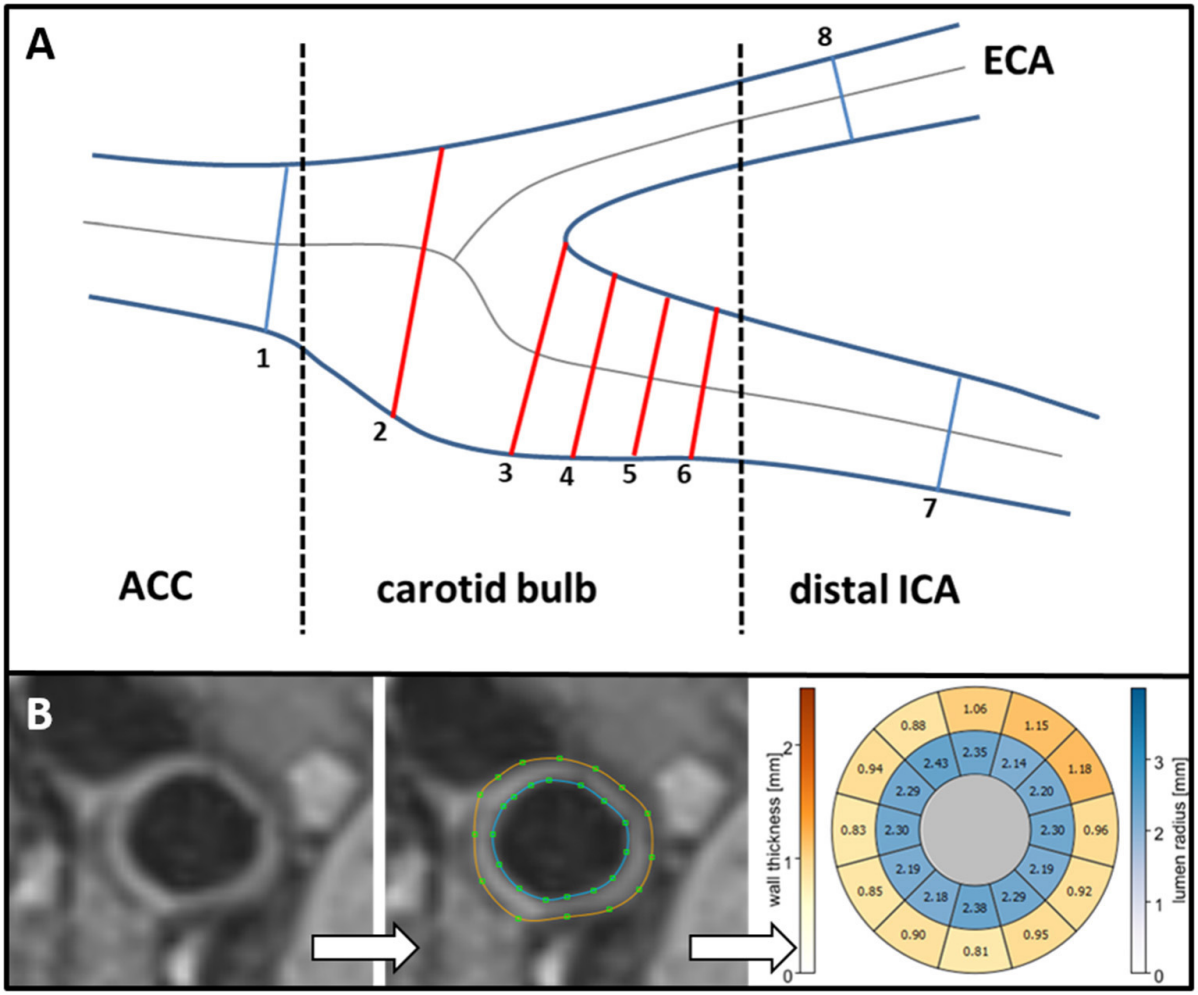
**(A)** Standardized position of the eight analysis planes. Planes 2–6 represent the carotid bulb. **(B)** T1-weighted cross-section illustrating carotid wall thickness (left). Manual segmentation of outer (brown line) and inner (blue line) vessel wall border in the cross-section (middle). Quantitative results of vessel lumen (inner ring labeled blue) and of wall thickness (outer ring labeled yellow to red) are displayed in a bulls-eye-plot comprising 12 evenly distributed wall segments (right). CCA, common carotid artery; ICA, internal carotid artery; ECA, external carotid artery.

For image processing, we imported data sets into a custom-made extension of the MEVISFlow research software [Fraunhofer MEVIS, Bremen, Germany (19)]. After noise filtering, correction for eddy currents, and velocity aliasing the software automatically created a carotid artery mask (3D phase-contrast MR angiography) and a centerline in each carotid bifurcation. As described previously (9), based on landmarks (flow diverter and ICA) eight cross-section planes were automatically positioned along the centerline in predefined locations and inter-plane distances. The first plane was positioned on the CCA centerline 1 cm below the flow diverter. The second plane was placed 0.5 cm below the flow diverter within the ICA bulb, planes 3–6 were placed along the ICA with the starting point at the flow diverter and oriented perpendicularly to the centerline with a spacing of 3 mm. Plane 7 was the most distal ICA plane outside the plaque and was positioned manually by the user. Plane 8 was placed automatically in the proximal external carotid artery (ECA). Each analysis plane consisted of 12 wall segments. Segment 1 was located at the posterior bulb. The remaining segments were numbered clockwise for the left and counterclockwise for the right carotid artery. This was performed in an identical fashion at baseline and follow-up to ensure measurement at the same location. Planes no. 2–6 represented the carotid bulb and were used for quantitative analysis of WSS and carotid wall thickness in our plane- and segment-based model (Figure 1). The required processing time of one 3D MRI dataset (bifurcation geometry, hemodynamics, wall thickness) was 45–60 min.

### Carotid Wall Thickness

After manual delineation of the inner and outer contours of the vessel wall in T1-weighted black-blood MR images wall thickness was automatically calculated (Figure 1B) (9). Definition of vessel wall contours was carried out by one reviewer (=observer 1) in the automatically generated 8 analysis planes of both carotid bifurcations. Observer 1 had performed these analyses in the identical fashion at baseline and follow-up and was blinded to patients' characteristics, values of wall thickness, carotid geometry, and hemodynamics at baseline.

### Geometry of the Carotid Bifurcation

**Figure 2:**
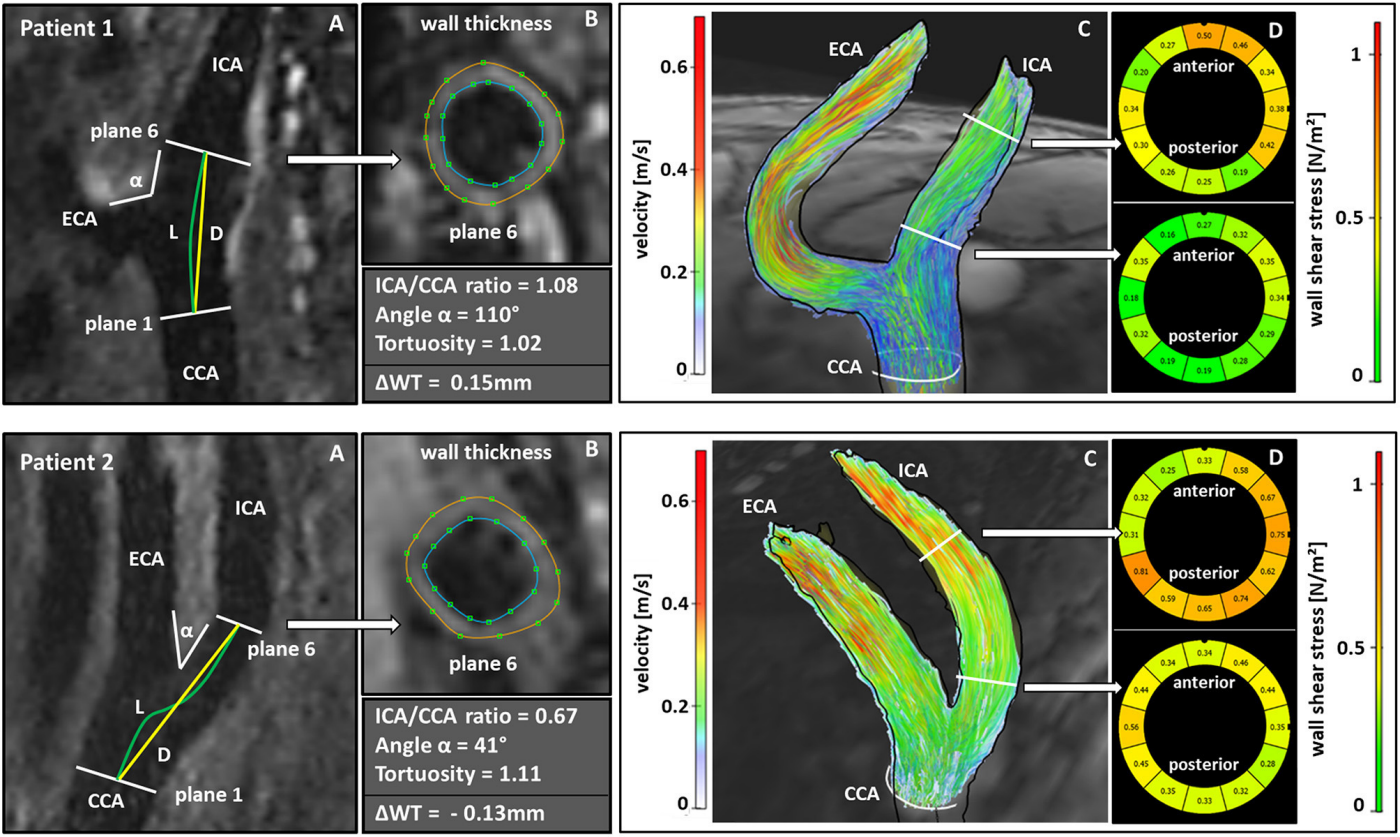
Comparison of geometric and hemodynamic properties associated with wall thickness progression **(Patient 1)** or plaque regression **(Patient 2)** during follow-up. Calculation of the ICA/CCA-ratio from the maximum diameters in plane 6 and plane1; L and D indicate the CCA-ICA distances along the lumen center (L) and the direct connection (D) for calculation of carotid tortuosity, α indicates bifurcation angle (A). Manual segmentation of wall thickness in the cross section corresponding to the white arrow (B). Streamlines represent absolute blood flow velocities in m/s derived from 4D flow MRI. (C). Wall shear stress analysis in the cross section corresponding to the white arrow (D). **(Patient 1)** showed an increase in wall thickness of 0.15 mm while **(Patient 2)** showed a decrease of wall thickness of 0.13 mm during follow-up. **(Patient 1)** had a higher ICA/CCA-ratio, a larger bifurcation angle, a lower tortuosity and showed lower wall shear stress values in the carotid bulb compared to **(Patient 2)** and compared to the values in the distal ICA. CCA, common carotid artery; ICA, internal carotid artery; ECA, external carotid artery; ΔWT, difference in wall thickness between follow-up and baseline examination.

Analysis of carotid bifurcation geometry was performed by observer 1. After manually defining CCA, ICA, and external carotid artery for centerline computation and based on the location of the flow diverter, geometry was analyzed based on the 3DTOF MR angiography as described before (9). Subsequently, (a) ICA/CCA-diameter ratio (maximum ICA diameter in plane 6 and CCA diameter in plane 1), (b) bifurcation angle (two tangential lines of the first 1 cm of the outer wall starting at the flow diverter), (c) CCA-ICA tortuosity (ratio of the direct line and the centerline connection of the CCA in plane 1 and plane 6 in the ICA) were calculated automatically (Figure 2A).

### Hemodynamic Parameters

A second reviewer (=observer 2) manually outlined the vessel-lumen boundary using the magnitude images of 4D flow MRI data and propagated them for each timeframe. Observer 2 had also performed this analysis at baseline in the identical fashion and was blinded to patients' characteristics, results of wall thickness, geometry, and hemodynamics at baseline. Absolute WSS (in N/m^2^ = 1 Pa) was time-averaged over the cardiac cycle and derived for each analysis plane and each vessel segment. Oscillatory shear index (OSI in %) was calculated as the degree of WSS inversion over the entire cardiac cycle as defined previously (5, 20, 21). Quantitative analysis was performed in planes 2–6 representing the carotid bulb (Figure 1).

### Accuracy of Wall Thickness Measurement

MRI was performed twice in five patients (i.e., in 10 carotid artery bifurcations) in order to determine the measurement accuracy of our outcome parameter “carotid wall thickness.” These patients were not part of our study cohort but fulfilled the same inclusion and exclusion criteria. Each patient underwent two MRI examinations on the same day in separate MRI sessions: patients were removed from the MR scanner after the first measurement, stood up, and were repositioned for the second MRI examination. We measured wall thickness analogously in the CCA (plane 1) and the carotid bulb (planes 2–6) of both sides resulting in a total of 60 planes that were available for the comparison of both MRI examinations. Both observers were experienced in the evaluation of MRI images with our software; observer 1 with more than 10 years of experience and observer 2 with 2 years of experience.

Observer 1 analyzed data of the first MR examination twice and data of the second MR examination to determine intra-observer agreement and reproducibility, respectively. Observer 1 performed analysis with a time interval of at least 7 days between analysis and was blinded to the other results. Observer 2 independently analyzed data of the first MR examination (=inter-observer agreement) and was blinded to the results of observer 1.

Intra-class correlation coefficients (ICC) were computed to assess inter- and intra-observer agreement and reproducibility. Values of 0.90 or higher for ICC represented excellent, 0.75–0.90 good, 0.50–0.75 fair, and <0.5 poor agreement (22).

### Statistical Analysis

Data are presented as mean and standard deviation or median [interquartile range (IQR)] for continuous variables and absolute frequencies and percentages for categorical variables. Depending on data distribution two-tailed *t*-tests or non-parametric tests were applied as appropriate for continuous variables.

In the primary analysis, we predicted follow-up wall thickness from baseline variables, most importantly geometric factors (ICA/CCA-diameter ratio, tortuosity, bifurcation angle). More specifically, we fitted an autoregressive linear mixed model with patient IDs as random intercepts to identify potential risk factors at baseline. We used a stepwise modeling strategy investigating the independent variables first and adjusted for other potential risk factors (age, male sex, side, new statin therapy, smoking, diabetes, and hyperlipidemia) in subsequent steps. Additionally, we performed the very same analytic strategy for hemodynamic parameters (absolute/systolic WSS, OSI). For all models, we performed a complete-case analysis. For internal model validation, we used *R*^2^ and examined the residuals. We inspected multi-collinearity using variance inflation factors and assessed spearman correlations between geometry and hemodynamics (Supplementary Figures 1, 2). All analyses are exploratory. Hence, *p*-values and 95% confidence intervals are descriptive and not corrected for multiple comparisons. This analysis was performed twice: first in the whole study cohort (group 1) and then after exclusion of all cases with ICA stenosis ≥10% (group 2) to exclude the potential influence of relevant inward remodeling.

## Results

### Study Population

**Figure 3:**
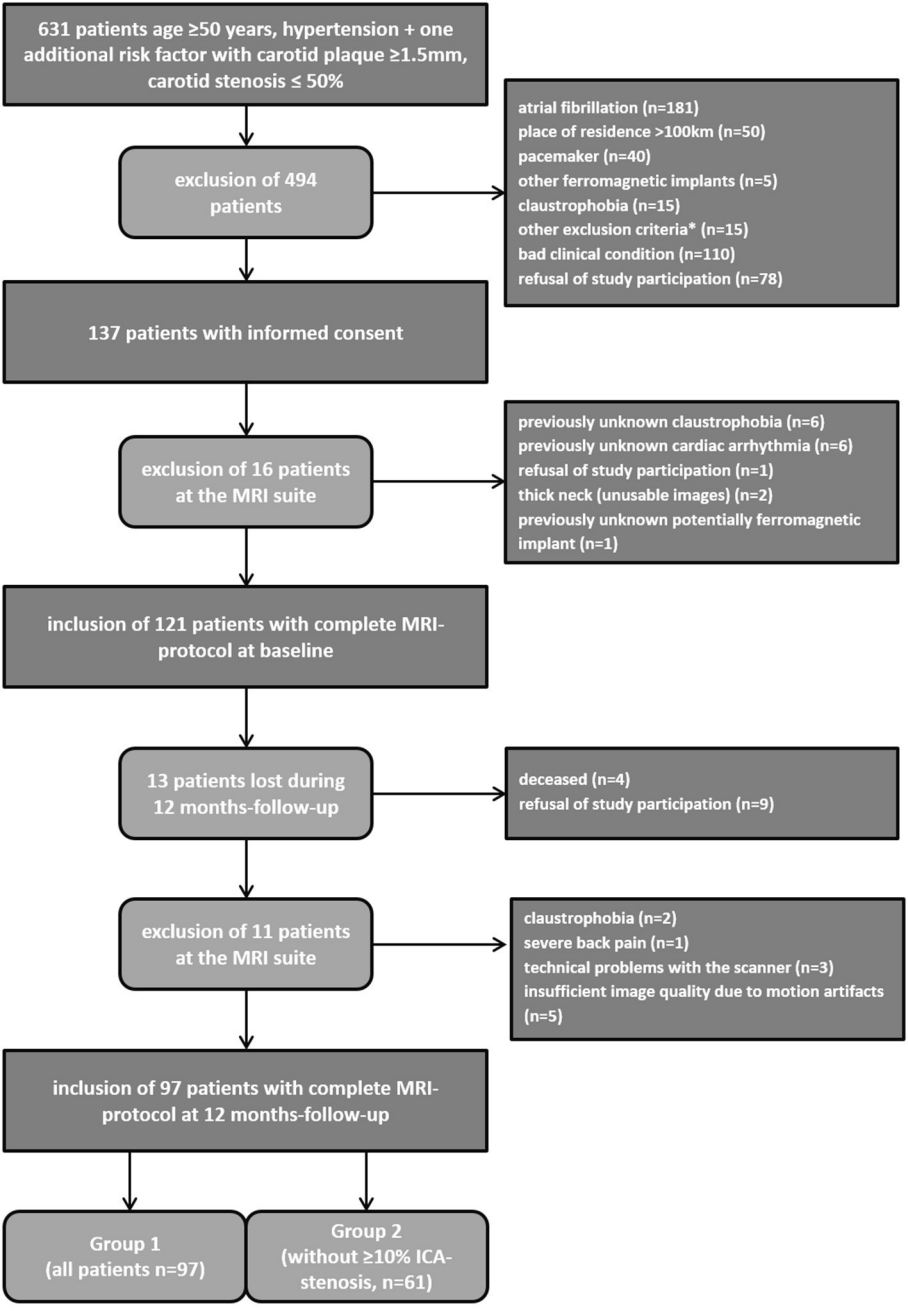
Recruitment algorithm of the study cohort. *To obese for MRI scanner (*n* = 6), expectation of life <2 years (*n* = 7), unable to lie due to back pain (*n* = 2).

24/121 patients studied at baseline were lost to follow-up, resulting in 97 patients that were available for the present analysis. Two of them underwent unilateral carotid surgery because of progression of ICA stenosis. Thus, follow-up MRI data was available in 97 patients and 192 carotid arteries (=group 1), respectively. Thirty-six patients had a degree of stenosis ≥10%. Accordingly, 61 patients and 122 carotid arteries without ICA stenosis ≥10% constituted group 2 (Figure 3).

Baseline characteristics are given in Table 1. The majority of our patients (89%) had an acute retinal or cerebral ischemia at baseline. The duration of follow-up was 374 ± 23.7 days. 37 patients (35.6%) received statin treatment before study inclusion, whereas the majority [65 patients (62.5%)] received statin therapy *de novo*. Two patients (1.9%) did not receive statins during follow-up.

**Table 1:** Baseline characteristics of the patients completing follow-up examinations.

**Characteristics**	**Group 1 (*n* = 97)**	**Group 2 (*n* = 61)**
Age (years)	70.1 (±8.4)	68.9 (±8.8)
Female sex—*n* (%)	29 (30.4)	22 (36.7)
Body mass index (kg/m^2^)	26.6 (±3.8)	26.1 (±3.9)
Hypertension—*n* (%)	97 (100.0)	61 (100)
Diabetes mellitus—*n* (%)	25 (26.2)	13 (21.7)
Peripheral artery disease—*n* (%)	8 (8.4)	2 (3.3)
Smoking habit—*n* (%)	24 (24.6)	17 (27.5)
Stroke/transient ischemic attack—*n* (%)	16 (16.8)	12 (19.2)
Coronary heart disease—*n* (%)	23 (23.6)	15 (24.2)
Hyperlipidemia—*n* (%)	66 (69.1)	38 (62.5)
Hb_A1c_ value (mmol/l)	40.1 (±12.6)	41.0 (±10.3)
LDL-cholesterol (mmol/l)	116.7 (± 50.6)	122.7 (±45.0)
ICA stenosis ≥10% to ≤ 50%—*n* (%)	36 (37.1)	–

*Group 1, entire study cohort; Group 2, patients without ICA stenosis ≥10%; LDL-cholesterol, low density lipoprotein—cholesterol; ICA, internal carotid artery*.

### Measurement Accuracy of Carotid Wall Thickness

Mean age of the five patients measured for the determination of carotid wall thickness accuracy was 71.2 ± 6.1 years and mean carotid wall thickness was 1.22 mm (IQR 1.10–1.35). Intra-class correlation coefficients (ICC) demonstrated excellent intra-observer agreement (ICC 0.95 [95% confidence interval (CI): 0.84–0.98]; coefficient of variance 0.05) and reproducibility (ICC = 0.93 [95% CI: 0.88–0.96]; coefficient of variance 0.05) for the measurement of carotid wall thickness. Inter-observer agreement was good (ICC = 0.82 [95% CI: 0.66–0.94]; coefficient of variance 0.08).

### Change of Carotid Wall Thickness During Follow-Up

**Figure 4:**
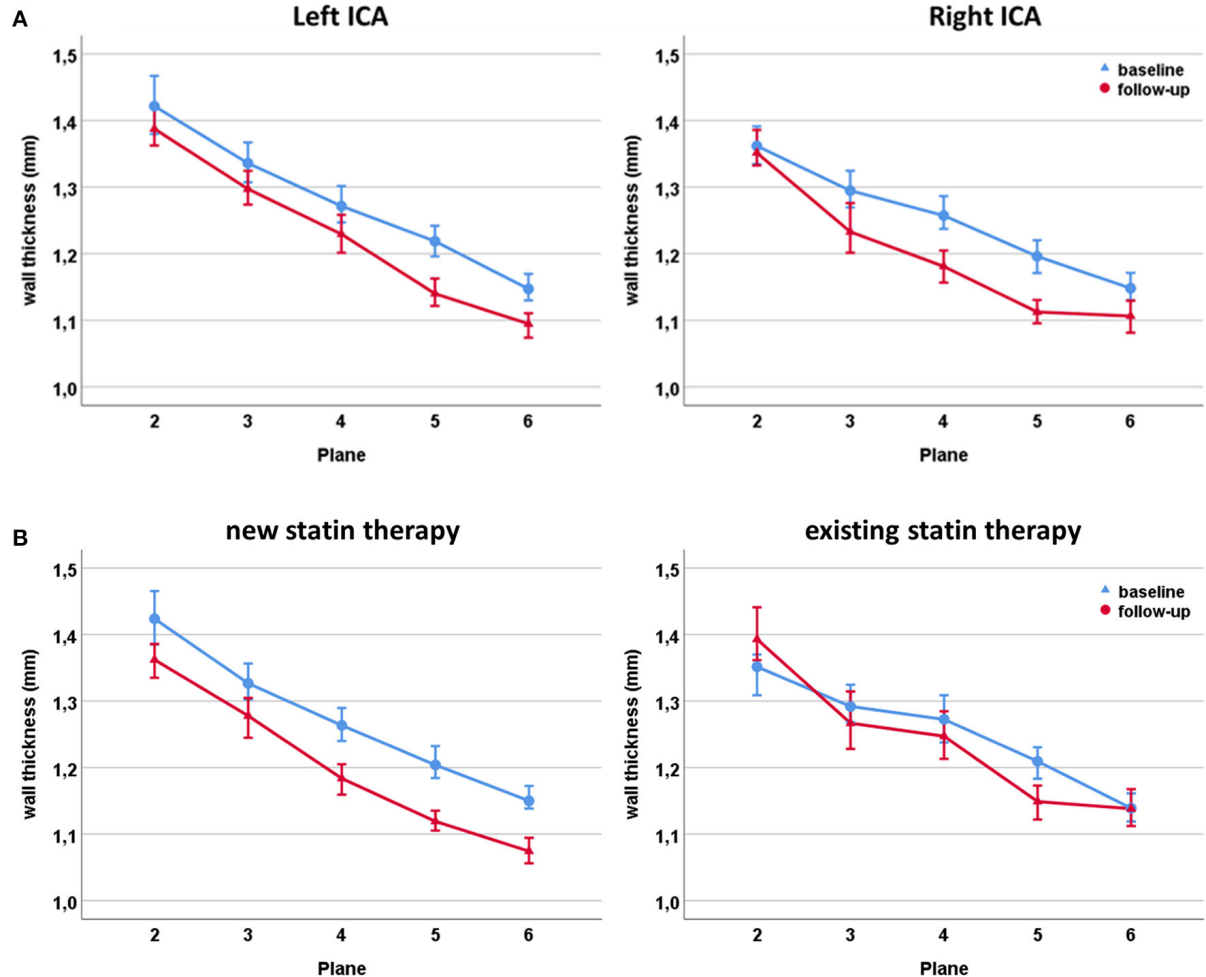
Changes of wall thickness during follow-up of the left and right carotid bulb **(A)**. Changes of wall thickness during follow-up of the carotid bulb in patients receiving *de novo* statin therapy (left) and patients with an existing statin therapy at study inclusion (right) **(B)**.

During follow-up, mean wall thickness in the carotid bulb (analysis planes 2–6) decreased from 1.25 mm (IQR: 1.06–1.57) at baseline to 1.21 mm (IQR: 1.02–1.55) in the overall study cohort (*p* < 0.001, Supplementary Table 1). Side specific differences between left and right carotid arteries are displayed in Figure 4A for each plane and all carotid bulbs in Supplementary Table 2.

Patients receiving *de novo* statin therapy at baseline showed a larger decrease in mean wall thickness (1.26 mm (IQR: 1.05–1.61) to 1.21 mm (IQR: 1.00–1.56); *p* < 0.001) compared to patients who were already on statins at study inclusion (1.24 mm (IQR 1.08–1.51) to 1.24 mm (IQR: 1.04–1.53); *p* = 0.02; Figure 4B and Supplementary Table 3).

**Figure 5:**
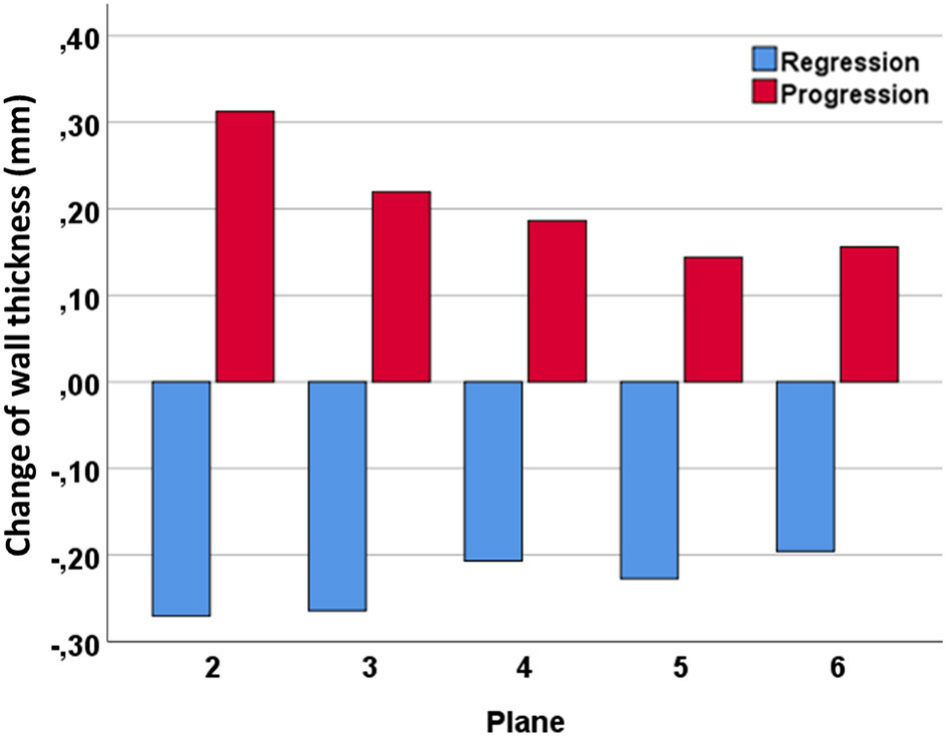
Distribution of progression and regression of carotid wall thickness during follow-up throughout the carotid bulb.

Figure 5 illustrates the distribution of progression and regression of wall thickness indicating that the largest changes occurred in the proximal bulb.

### Independent Predictors of Carotid Wall Thickness in All Patients (Group 1)

Results of the final model for group 1 (97 patients; 192 carotid bifurcations) regarding the effect of geometry at baseline on carotid wall thickness at follow-up (=dependent variable/outcome parameter) are given in Table 2. Stepwise models for group 1 are shown in Supplementary Tables 4, 6. An increase of carotid tortuosity was significantly associated with decreased wall thickness at follow-up even after adjusting for age, sex, baseline wall thickness, new statin therapy, and cardiovascular risk factors (regression coefficient −0.52 [95% CI: −0.76 to −0.28], *p* < 0.001). Age (0.01 [95% CI: 0.00–0.01], *p* < 0.001) and hyperlipidemia (0.08 [95% CI: 0.00–0.15], *p* = 0.037) independently predicted wall thickness progression at follow-up. Moreover, regression of carotid wall thickness was stronger on the right side (−0.02 [95% CI: −0.04 to −0.00], *p* = 0.045).

**Table 2:** Final models of multivariate autoregressive analysis for geometric and hemodynamic parameters and their independent prediction of wall thickness at follow-up as the dependent variable.

	**Carotid geometry**				**Carotid hemodynamics**			
	**Group 1**		**Group 2**		**Group 1**		**Group 2**	
**Predictors**	**Estimates**	**95%-CI**	**Estimates**	**95%-CI**	**Estimates**	**95%-CI**	**Estimates**	**95%-CI**
(Intercept)	0.26	−0.16 to 0.67	−0.20	−0.69 to 0.28	−0.27	−0.59 to 0.04	0.00	−0.31 to 0.32
Baseline wall thickness	0.71^*^	0.70 to 0.73	0.59^*^	0.57 to 0.61	0.71^*^	0.70 to 0.73	0.59^*^	0.57 to 0.61
ICA/CCA-ratio	0.02	−0.06 to 0.11	0.49^*^	0.39 to 0.60	–	–	–	–
Tortuosity	−0.52^*^	−0.76 to −0.28	−0.30^‡^	−0.59 to −0.01	–	–	–	–
Bifurcation angle	0.01	−0.01 to 0.02	0.04^†^	0.02 to 0.06	–	–	–	–
Oscillatory shear index (OSI)	–	–	–	–	0.02	−0.08 to 0.11	0.03	−0.06 to 0.13
Wall shear stress (WSS)	–	–	–	–	0.00	−0.02 to 0.02	−0.03^†^	−0.05 to −0.01
Age	0.01^*^	0.00 to 0.01	0.01^†^	0.00 to 0.01	0.01	0.00 to 0.01	0.01^†^	0.00 to 0.01
Sex (male)^a^	0.04	−0.04 to 0.11	0.04	−0.04 to 0.13	0.04	−0.03 to 0.11	0.06	−0.02 to 0.13
Carotid artery side (right)^a^	−0.03^†^	−0.05 to −0.01	−0.02	−0.04 to 0.00	−0.02^‡^	−0.04 to −0.00	−0.06^*^	−0.07 to −0.04
New statin therapy (yes)^a^	−0.02	−0.09 to 0.05	0.01	−0.07 to 0.10	−0.01	−0.08 to 0.05	0.06	−0.02 to 0.13
Smoking (yes)^a^	0.01	−0.07 to 0.09	0.01	−0.09 to 0.10	0.02	−0.07 to 0.10	−0.02	−0.10 to 0.07
Diabetes (yes)^a^	0.04	−0.04 to 0.12	0.04	−0.07 to 0.14	0.04	−0.04 to 0.11	0.02	−0.08 to 0.11
Hyperlipidemia (yes)^a^	0.08^‡^	0.00 to 0.15	0.05	−0.04 to 0.13	0.08^‡^	0.00 to 0.15	0.03	−0.04 to 0.11
Marginal *R*^2^/Conditional *R*^2^	0.541/0.592		0.413/0.503		0.541/0.591		0.405/0.479	

*Group 1 = total study cohort (n = 97), group 2 = patients without ICA stenosis ≥10% (n = 61)*.

a *Reference categories: female (sex), left (side), no (new statin therapy, smoking, diabetes, and hyperlipidemia)*;

* *p <0.001*,

† *p <0.01, and*

‡ *p <0.05*.

However, we neither found a significant association of ICA/CCA-ratio, bifurcation angle, WSS, and OSI nor new statin therapy with carotid wall thickness at follow-up.

### Independent Predictors of Carotid Wall Thickness in Patients Without ICA Stenosis (Group 2)

Results of the final model for group 2 (61 patients, 122 carotid arteries) regarding the effect of geometry at baseline on carotid wall thickness at follow-up (=dependent variable/outcome parameter) are given in Table 2 (stepwise models for group 2 are shown in Supplementary Tables 5, 7).

Interestingly, this analysis showed a significant positive association of ICA/CCA-ratio (0.49, 95% CI [0.39–0.60], *p* < 0.001), bifurcation angle (0.04, 95% CI [0.02–0.06], *p* = 0.001), baseline wall thickness (0.59, 95% CI [0.57–0.61], *p* < 0.001), and age (0.01, 95% CI [−0.00 to 0.01], *p* = 0.001) at baseline with carotid wall thickness at follow-up. A significant inverse association was found for tortuosity (−0.30, 95% CI [−0.59 to −0.01], *p* = 0.040) and absolute WSS (−0.03, 95% CI [−0.05 to −0.01], *p* = 0.010) with increased wall thickness at follow-up. These associations remained significant after adjusting for age, sex, right/left side, new statin therapy, and cardiovascular risk factors. Key findings are illustrated in two patients in Figure 2.

## Discussion

In the present study, we investigated the impact of both carotid bifurcation geometry and wall shear stress on carotid wall thickness in 97 patients with high cardiovascular risk during a 12-month period. To our knowledge, this is the first study that investigated this issue longitudinally in a larger high-risk cohort based on a 3D multi-contrast and 4D flow MRI protocol *in vivo* without the need of additional computational analysis. Measurement accuracy of carotid wall thickness was excellent and thus underlines the reliability of our findings.

In the whole study cohort (group 1), larger carotid tortuosity representing a less straight course of the ICA was an independent predictor for the regression and age and hyperlipidemia were independent predictors for the progression of carotid wall thickness. In patients without ICA stenosis ≥10% (group 2) a larger bulb of the ICA, a greater bifurcation angle, and lower WSS independently predicted carotid wall thickness progression over time. And again, larger carotid tortuosity resulted in a regression of wall thickness.

### Impact of Geometry on Carotid Wall Thickness

One of the main findings is the independent prediction of an increased ICA/CCA-ratio for the progression of wall thickness in patients without ICA stenosis (group 2). Previous CFD and *in vivo* studies in volunteers and patients have demonstrated that the local expansion of the ICA bulb promotes “disturbed flow,” i.e., a typical pattern of low and oscillatory wall shear stress (5, 6, 9). Accordingly, geometry obviously produces a circumscribed atherosclerosis-prone environment in this part of the carotid bifurcation. This is in line with Bijari et al. (7) concluding that flare, a parameter similar to the ICA/CCA-ratio and describing a large carotid bifurcation, is a predictor of wall thickness in earlier stages of atherosclerosis in patients without inward remodeling. Compatible with this study we were not able to show this association in all patients, i.e. including those with ≥10% ICA stenosis (group 1), which is most likely due to relevant inward remodeling in this group masking this effect.

These findings may appear contradictory to the results of cross-sectional studies in patients with atherosclerotic lesions (8, 9). Phan et al. (8) reported an independent association of reduced ICA radius and ICA/CCA-ratio with the development of carotid stenosis. In addition, our baseline analysis of this study cohort demonstrated that a decreased ICA/CCA-ratio was an independent predictor of increased carotid wall thickness (9). However, patients with larger carotid plaques or stenosis, i.e. with advanced atherosclerosis, show a consecutive reduction of the ICA bulb diameter by inward remodeling and thus the inverse association: a smaller diameter due to stenosis is related to a thicker wall thickness. Accordingly, the comparison of the large ICA bulb with future wall thickening is not possible anymore.

A higher bifurcation angle predicted increased wall thickening over time in carotid bifurcations after exclusion of ≥10% ICA stenosis. Previous CFD-studies (23) demonstrated the impact of the bifurcation angle on “disturbed flow” in idealized geometries but former cross-sectional studies in healthy volunteers (5, 6) were not able to show a significant correlation with low WSS. We neither detected a correlation of increased bifurcation angle with wall thickness in our baseline analysis (9) while Phan et al. (8) reported an independent positive association of ICA angle, which is a subset of the bifurcation angle, with carotid stenosis. Thus, the true impact of the bifurcation angle on changes in local hemodynamics and finally wall thickness remains unclear and requires further investigation.

Finally, a higher carotid tortuosity at baseline independently predicted a regression of wall thickness in both groups. This suggests an athero-protective effect of such geometry on the local distribution of blood flow resulting in an increased helical flow pattern. This is in line with former CFD studies in carotid and coronary arteries (24–26) demonstrating a potentially athero-protective effect of increased helical flow through the suppression of “disturbed flow,” generation of relatively uniform WSS and prevention of the interaction of critical low WSS or high oscillatory shear stress with the arterial wall. Furthermore, there is evidence that increased helical flow reduces the transfer of atherogenic particles such as low-density lipoproteins into the vessel wall (27). Finally, stents with a helical shape and thus higher tortuosity show lower restenosis rates by inducing a laminar swirling flow that elevates athero-protective WSS (28). Based on our results it seems that a higher tortuosity has also protective properties in patients with preexisting carotid stenosis. This may be explained by the fact that patients with more advanced stages of atherosclerosis have more elongated vessels through long-term exposure of arterial hypertension, leading to elongation, coiling, and ultimately increased carotid tortuosity (29).

In summary, in this prospective and longitudinal patient study, we were able to confirm the influence of carotid geometry on wall thickness, in particular when relevant inward remodeling such as in ≥10% ICA stenosis was excluded (7).

### Impact of Wall Shear Stress on Carotid Wall Thickness

The “geometry risk hypothesis” proposes that disturbed flow induces a pro-atherogenic environment. Fittingly, we found that low WSS at baseline was an independent predictor of increased carotid wall thickness over time. This effect was present after exclusion of ≥10% ICA stenosis and thus relevant inward remodeling. The negative effect of low WSS was previously demonstrated in animal models (4). However, this is the first larger study in patients that was able to detect this independent role of WSS on carotid atherosclerosis based on 3D data and independent from additional computational fluid analysis.

Cibis et al. (12) had also detected an increased carotid wall thickness during a 4-year follow-up in low WSS areas. However, they studied only 14 patients and required CFD analysis. A two-dimensional ultrasound study in 48 patients showed plaque progression in low WSS areas during a 12-year follow-up (13). Finally, another small study used MRI in combination with CFD, included 13 patients after carotid surgery and studied restenosis rates over 5 years (14). The two patients with the highest flare and therefore largest exposure to low WSS developed significant restenosis during follow-up.

The independent predictive values of low WSS on wall thickness progression became significant after excluding ≥10% ICA stenosis, which may be explained by the hypothesis that low WSS and high OSI play an important role in initiating the atherosclerotic cascade (30, 31). By contrast, in more pronounced stages of atherosclerosis such as ≥10% ICA stenosis, other factors such as high WSS or plaque surface pressure may play a more important role as a trigger for the progression and finally rupture of atheroma (32, 33).

### Impact of Statin Treatment on Carotid Wall Thickness

We measured a slight decrease in overall carotid wall thickness during follow-up, which is most probably due to the initiation of new statin therapy at study inclusion in two-thirds of our patients because these patients showed the strongest regression of wall thickness. Most patients had acute retinal or cerebral ischemia at the time of recruitment and thus received statins as an essential part of future stroke prevention. Our interpretation is supported by previous studies that demonstrated a regression of carotid wall thickness up to 8% after 12 months and even 20% after 24 months under statin treatment (34). Possibly, the effect of statins may have masked an even stronger correlation of geometrical and hemodynamical parameters on carotid wall thickness changes even if we could not identify an independent effect in statistical analysis in our cohort.

### Limitations

The relative changes in carotid wall thickness during follow-up were small and the overall reduction of wall thickness was probably due to the new statin therapy in the majority of patients. Additionally, an observation period of 12-months is relatively short to detect changes in carotid wall thickness, even in high-risk patients. Thus, an extension of the follow-up period under stable therapy with statins is certainly reasonable and therefore planned by our group.

As we examined a high-risk cohort with preexisting atherosclerotic changes we cannot completely rule out a certain influence of inward remodeling on our results even after exclusion of patients with >10% degree of stenosis. Future studies in cohorts free of atherosclerosis might produce even more significant results regarding the influence of geometry and hemodynamic parameters on progression of carotid wall thickness.

WSS analysis using 4D flow MRI *in vivo* represents a very realistic assessment of the distribution of WSS, allows for a reliable analysis of relative differences but systematically underestimates absolute values (35). Thus, a further increase of both spatial and temporal resolution of 4D flow MRI using new acceleration techniques such as compressed sensing may be helpful to improve measurement accuracy and the evaluation of the independent role of WSS *in vivo*.

Carotid wall thickness is the clinically relevant outcome parameter for stroke as it finally leads to carotid artery stenosis and plaque rupture. Future longitudinal studies should also examine the influence of geometry and WSS on plaque composition and vulnerability as clinical outcome parameters because they are in closer relation to plaque rupture and imminent cerebrovascular events than wall thickness.

## Conclusion

In this longitudinal study, we were able to demonstrate the independent influence of both carotid geometry and hemodynamics on the progression of wall thickness *in vivo*. A large carotid bulb, a large bifurcation angle and low WSS were independent predictors for the progression of atherosclerotic wall changes independent of cardiovascular risk factors in ≤ 10% ICA stenosis. In contrast, a high carotid tortuosity seems to protect the ICA from atherosclerosis, which is probably due to the consecutive and beneficial helical flow pattern. Our results and the presented carotid biomarkers may be important for the future management of patients with carotid atherosclerosis. They might lead to an intensified monitoring and conservative treatment in certain individual geometrical and hemodynamical conditions. In addition, future techniques for carotid surgery or stent design may benefit from optimal shaping of the ICA bulb based on our findings.

## Data Availability Statement

The raw data supporting the conclusions of this article will be made available by the authors, without undue reservation.

## Ethics Statement

The studies involving human participants were reviewed and approved by Ethics Committee of the Albert-Ludwigs University of Freiburg, Germany. The patients/participants provided their written informed consent to participate in this study.

## Author Contributions

CS was involved in the design of the study, recruited participants, performed data analysis, interpreted the data, and drafted the manuscript. AK, UL, and JH developed the MRI-protocol and revised the manuscript. MH, LK, and AHe developed our 4D flow MRI-data analysis software and revised the manuscript. MT and GK performed the statistical data analysis and revised the manuscript. AHa designed the study, interpreted the data, drafted, and revised the manuscript. All authors read and approved the final manuscript.

## Funding

CS and AHa were supported by Deutsche Forschungsgemeinschaft (DFG) grant DFG HA 5399/5-1. AK and UL were supported by DFG grant HE 1875/29-1. AHe, LK, and MH were supported by DFG grants HA 5399/5-1, HE 7312/4-1, and HE 1875/29-1. The article processing charge was funded by the Baden-Wuerttemberg Ministry of Science, Research, and Art and the University of Freiburg in the funding programme Open Access Publishing.

## Conflict of Interest

The authors declare that the research was conducted in the absence of any commercial or financial relationships that could be construed as a potential conflict of interest.

## Publisher's Note

All claims expressed in this article are solely those of the authors and do not necessarily represent those of their affiliated organizations, or those of the publisher, the editors and the reviewers. Any product that may be evaluated in this article, or claim that may be made by its manufacturer, is not guaranteed or endorsed by the publisher.
