# Supplementary material for: Carotid Geometry and Wall Shear Stress Independently Predict Increased Wall Thickness—A Longitudinal 3D MRI Study in High-Risk Patients
Source: Front Cardiovasc Med. 2021 Oct 26;8:723860. doi: 10.3389/fcvm.2021.723860 (PMC8576112; doi:10.3389/fcvm.2021.723860)
Supplement: Supplementary file 1 [file Data_Sheet_1.docx]

**Supplemental material**

**Carotid geometry and wall shear stress independently predict increased wall thickness– a longitudinal 3D MRI study**

Christoph Strecker^1^, Axel Joachim Krafft^2^, Lilli Kaufhold^3,4^, Markus Hüllebrandt^3,4^, Martin Treppner^5^, Ute Ludwig^2^, Göran Köber^5^, Anja Hennemuth^3,4^, Jürgen Hennig^2^ and Andreas Harloff^1^

^1^ Department of Neurology and Neurophysiology, Medical Center - University of Freiburg, Faculty of Medicine, University of Freiburg, Germany

## ^2^ Department of Radiology - Medical Physics, Medical Center - University of Freiburg, Faculty of Medicine, University of Freiburg, Freiburg, Germany

^3^ Fraunhofer MEVIS, Bremen, Germany

^4^ Institute for Imaging Science and Computational Modelling in Cardiovascular Medicine, Charité-Universitätsmedizin Berlin, Berlin, Germany

^5^ Institute of Medical Biometry and Statistics, Faculty of Medicine and Medical Center, University of Freiburg, Germany

**Corresponding author:**

Christoph Strecker, MD

Department of Neurology and Neurophysiology

Medical Center - University of Freiburg

Breisacherstrasse 64

79106 Freiburg

Germany

Phone: 0049-761-270 50010

**Detailed description of MRI-scanning protocol**

An isotropic spatial resolution of 0.6mm^3^ was used for all sequences except for 3D TOF MR angiography (0.5x0.5x0.6mm^3^). Other sequence parameters were:

- 3D-TOF*:* TR/TE=21/3.3ms, axial field of view (FOV)=180x135mm² (read x phase), matrix 384x290 (read x phase), longitudinal coverage=910mm, flip angle=20°, pixel bandwidth (BW)= 250Hz/Px, total acquisition time (TA)=4:32min.
- T1-weighted SPACE*:* TR/TE=900/26ms, axial FOV=154x38 mm^2^ (read x phase), matrix 256x128 (read x phase), longitudinal coverage=480mm, BW=405 Hz/Px, echo train length (ETL)=36, TA=7:32min.
- T2-weighted SPACE*:* TR/TE=2000/159ms, axial FOV=154x38 mm^2^ (read x phase), matrix 256x128 (read x phase), longitudinal coverage=480mm, BW=405Hz/Px, ETL=61, TA=7:22min.
- PD-weighted SPACE*:* TR/TE=1900/26ms, axial FOV=154x38 mm^2^ (read x phase), matrix 256x128 (read x phase), longitudinal coverage=480mm, BW=405Hz/Px, ETL=61, TA=8:04min.
- 4D flow MRI: spatial/temporal resolution=0.8mm^3^/52.8ms, TR/TE=52.8/3.9ms, axial FOV=140x140mm^2^, matrix 192x192 (read x phase), longitudinal coverage=510mm, flip angle=12°, PEAK GRAPPA acceleration factor=5, slice thickness=0.8mm, BW=460 Hz/Px, VENC (in-plane)=0.6m/s, VENC (through plane)=1.0m/s.

**Tables I-VII and Figures I-II**

|  | **Baseline** | **Follow up** | **p-value** |
| --- | --- | --- | --- |
| **Wall thickness (mm)** | 1.25 (1.06-1.57) | 1.21 (1.01-1.55) | 0.001 |
| **WSS (N/m²)** | 0.52 (0.36-0.76) | 0.52 (0.37-0.75) | 0.340 |
| **OSI (%)** | 14.0 (6.3-22.6) | 9.6 (2.1-19.1) | 0.001 |
| **ICA/CCA-Ratio** | 0.73 (0.62-0.87) | 0.74 (0.65-0.88) | 0.001 |
| **Bifurcation angle (°)** | 45.0 (31.6-67.5) | 46.1 (30.7-62.7) | 0.530 |
| **Tortuosity** | 1.05 (1.02-1.09) | 1.06 (1.03-1.10) | 0.001 |

**Table I:** Descriptive statistics (median (IQR)) of wall thickness, hemodynamic parameters (WSS=wall shear stress; OSI=oscillatory shear index) and geometric parameters (ICA/CCA-ratio, bifurcation angle and tortuosity) for the overall study cohort between baseline and follow-up.

| ***Baseline*** | **Right ICA** | **Left ICA** | **p-value** |
| --- | --- | --- | --- |
| **Wall thickness (mm)** | 1.24 (1.05-1.54) | 1.27 (1.08-1.60) | 0.001 |
| **WSS (N/m²)** | 0.50 (0.35-0.73) | 0.53 (0.38-0.80) | 0.001 |
| **OSI (%)** | 13.4 (5.8-22.3) | 14.6 (6.7-22.9) | 0.001 |
| **ICA/CCA-Ratio** | 0.73 (0.62-0.83) | 0.76 (0.64-0.89) | 0.001 |
| **Bifurcation angle (°)** | 43.6 (29.9-65.7) | 48.15 (37.5-70.6) | 0.001 |
| **Tortuosity** | 1.04 (1.03-1.08) | 1.06 (1.03-1.11) | 0.001 |

| ***Follow up*** | **Right ICA** | **Left ICA** | **p-value** |
| --- | --- | --- | --- |
| **Wall thickness (mm)** | 1.20 (1.00-1.51) | 1.24 (1.03-1.58) | 0.001 |
| **WSS (N/m²)** | 0.50 (0.36-0.71) | 0.55 (0.40-0.80) | 0.001 |
| **OSI (%)** | 8.3 (1.8-18.3) | 10.8 (2.8-19.8) | 0.001 |
| **ICA/CCA-Ratio** | 0.71 (0.63-0.84) | 0.78 (0.70.0.89) | 0.001 |
| **Bifurcation angle (°)** | 43.8 (28.9-60.4) | 51.0 (34.7-67.0) | 0.001 |
| **Tortuosity** | 1.05 (1.03-1.10) | 1.07 (1.04-1.13) | 0.001 |

**Table II:** Descriptive statistics (median (IQR)) of wall thickness, hemodynamic parameters (WSS=wall shear stress; OSI=oscillatory shear index) and geometric parameters (ICA/CCA-ratio, bifurcation angle and tortuosity) between the left and right carotid artery.

| ***Baseline*** | **de novo statin therapy** | **preexisting statin therapy** | **p-value** |
| --- | --- | --- | --- |
| **Wall thickness (mm)** | 1.26 (1.05-1.61) | 1.24 (1.08-1.51) | 0.033 |
| **WSS (N/m²)** | 0.53 (0.37-0.80) | 0.49 (0.35-0.72) | 0.001 |
| **OSI (%)** | 13.9 (6.2-22.6) | 14.4 (6.5-22.6) | 0.184 |
| **ICA/CCA-Ratio** | 0.72 (0.62-0.83) | 0.80 (0.64-0.91) | 0.001 |
| **Bifurcation angle (°)** | 50.8 (32.4-69.6) | 40.4 (30.4-57.9) | 0.001 |
| **Tortuosity** | 1.05 (1.03-1.09) | 1.05 (1.03-1.08) | 0.001 |

| ***Follow up*** | **de novo statin therapy** | **preexisting statin therapy** | **p-value** |
| --- | --- | --- | --- |
| **Wall thickness (mm)** | 1.21 (1.00-1.56) | 1.24 (1.04-1.53) | 0.001 |
| **WSS (N/m²)** | 0.53 (0.38-0.76) | 0.52 (0.37-0.75) | 0.001 |
| **OSI (%)** | 9.8 (2.7-19.2) | 9.1 (0.9-18.7) | 0.172 |
| **ICA/CCA-Ratio** | 0.72 (0.64-0.85) | 0.83 (0.69-0.89) | 0.001 |
| **Bifurcation angle (°)** | 47.9 (34.0-65.7) | 41.1 (29.5-60.5) | 0.001 |
| **Tortuosity** | 1.07 (1.03-1.11) | 1.05 (1.03-1.08) | 0.001 |

**Table III:** Descriptive statistics (median (IQR)) of wall thickness, hemodynamic parameters (WSS=wall shear stress; OSI=oscillatory shear index) and geometric parameters (ICA/CCA-ratio, bifurcation angle and tortuosity) between patients receiving new statin therapy and preexisting statin therapy before study inclusion (C).

|  | Model 1 | | | Model 2 | | | Model 3 | | |
| --- | --- | --- | --- | --- | --- | --- | --- | --- | --- |
| **Predictors** | Estimates | CI | p | Estimates | CI | p | Estimates | CI | p |
| **(Intercept)** | 0.78 | 0.53 – 1.03 | <0.001 | 0.17 | -0.21 – 0.55 | 0.387 | 0.26 | -0.16 – 0.67 | 0.225 |
| **Baseline wall thickness** | 0.71 | 0.70 – 0.73 | <0.001 | 0.71 | 0.70 – 0.73 | <0.001 | 0.71 | 0.70 – 0.73 | <0.001 |
| **ICA/CCA ratio** | 0.04 | -0.05 – 0.12 | 0.396 | 0.04 | -0.04 – 0.13 | 0.333 | 0.02 | -0.06 – 0.11 | 0.582 |
| **Tortuosity** | -0.41 | -0.64 – -0.17 | 0.001 | -0.42 | -0.65 – -0.19 | <0.001 | -0.52 | -0.76 – -0.28 | <0.001 |
| **Bifurcation angle** | 0.01 | -0.01 – 0.02 | 0.324 | 0.01 | -0.01 – 0.02 | 0.308 | 0.01 | -0.01 – 0.02 | 0.355 |
| **Age** |  |  |  | 0.01 | 0.00 – 0.01 | <0.001 | 0.01 | 0.00 – 0.01 | <0.001 |
| **Sex (male)^a^** |  |  |  | 0.04 | -0.03 – 0.11 | 0.286 | 0.04 | -0.04 – 0.11 | 0.344 |
| **Carotid artery side (right) ^a^** |  |  |  |  |  |  | -0.03 | -0.05 – -0.01 | 0.002 |
| **New statin therapy (yes) ^a^** |  |  |  |  |  |  | -0.02 | -0.09 – 0.05 | 0.580 |
| **Smoking (yes) ^a^** |  |  |  |  |  |  | 0.01 | -0.07 – 0.09 | 0.797 |
| **Diabetes (yes) ^a^** |  |  |  |  |  |  | 0.04 | -0.04 – 0.12 | 0.351 |
| **Hyperlipidemia (yes) ^a^** |  |  |  |  |  |  | 0.08 | 0.00 – 0.15 | 0.037 |
| **Marginal R2 / Conditional R2** | 0.520 / 0.583 | | | 0.539 / 0.591 | | | 0.541 / 0.592 | | |

**Table IV:** Stepwise autoregressive linear mixed models for geometric parameters with wall thickness at follow-up as the dependent variable in the whole study cohort (=group 1).

^a^ Reference categories: female (sex), left (side), no (new statin therapy, smoking, diabetes, hyperlipidemia)

|  | Model 1 | | | Model 2 | | | Model 3 | | |
| --- | --- | --- | --- | --- | --- | --- | --- | --- | --- |
| **Predictors** | Estimates | CI | p | Estimates | CI | p | Estimates | CI | p |
| **(Intercept)** | 0.36 | 0.05 – 0.67 | 0.024 | -0.28 | -0.72 – 0.16 | 0.218 | -0.20 | -0.69 – 0.28 | 0.412 |
| **Baseline wall thickness** | 0.59 | 0.57 – 0.61 | <0.001 | 0.59 | 0.57 – 0.61 | <0.001 | 0.59 | 0.57 – 0.61 | <0.001 |
| **ICA/CCA ratio** | 0.52 | 0.43 – 0.62 | <0.001 | 0.52 | 0.43 – 0.62 | <0.001 | 0.49 | 0.39 – 0.60 | <0.001 |
| **Tortuosity** | -0.26 | -0.54 – 0.03 | 0.078 | -0.25 | -0.53 – 0.03 | 0.085 | -0.30 | -0.59 – -0.01 | 0.040 |
| **Bifurcation angle** | 0.04 | 0.02 – 0.06 | <0.001 | 0.04 | 0.02 – 0.06 | <0.001 | 0.04 | 0.02 – 0.06 | 0.001 |
| **Age** |  |  |  | 0.01 | 0.00 – 0.01 | <0.001 | 0.01 | 0.00 – 0.01 | 0.001 |
| **Sex (male)^a^** |  |  |  | 0.05 | -0.03 – 0.13 | 0.217 | 0.04 | -0.04 – 0.13 | 0.308 |
| **Carotid artery side (right) ^a^** |  |  |  |  |  |  | -0.02 | -0.04 – 0.00 | 0.085 |
| **New statin therapy (yes) ^a^** |  |  |  |  |  |  | 0.01 | -0.07 – 0.10 | 0.738 |
| **Smoking (yes) ^a^** |  |  |  |  |  |  | 0.01 | -0.09 – 0.10 | 0.859 |
| **Diabetes (yes) ^a^** |  |  |  |  |  |  | 0.04 | -0.07 – 0.14 | 0.478 |
| **Hyperlipidemia (yes) ^a^** |  |  |  |  |  |  | 0.05 | -0.04 – 0.13 | 0.266 |
| **Marginal R2 / Conditional R2** | 0.384 / 0.495 | | | 0.416 / 0.504 | | | 0.413 / 0.503 | | |

**Table V:** Stepwise autoregressive linear mixed models for geometric parameters with wall thickness at follow-up as the dependent variable in patients without ICA stenosis ≥10 % (=group 2).

^a^ Reference categories: female (sex), left (side), no (new statin therapy, smoking, diabetes, hyperlipidemia)

|  | Model 1 | | | Model 2 | | | Model 3 | | |
| --- | --- | --- | --- | --- | --- | --- | --- | --- | --- |
| **Predictors** | Estimates | CI | p | Estimates | CI | p | Estimates | CI | p |
| **(Intercept)** | 0.37 | 0.32 – 0.41 | <0.001 | -0.23 | -0.52 – 0.06 | 0.118 | -0.27 | -0.59 – 0.04 | 0.091 |
| **Baseline wall thickness** | 0.71 | 0.70 – 0.73 | <0.001 | 0.71 | 0.70 – 0.73 | <0.001 | 0.71 | 0.70 – 0.73 | <0.001 |
| **Oscillatory shear index (OSI)** | 0.02 | -0.08 – 0.11 | 0.694 | 0.02 | -0.08 – 0.11 | 0.743 | 0.02 | -0.08 – 0.11 | 0.745 |
| **Wall shear stress (WSS)** | 0.01 | -0.01 – 0.03 | 0.576 | 0.01 | -0.01 – 0.03 | 0.574 | 0.00 | -0.02 – 0.02 | 0.688 |
| **Age** |  |  |  | 0.01 | 0.00 – 0.01 | <0.001 | 0.01 | 0.00 – 0.01 | <0.001 |
| **Sex (male)^a^** |  |  |  | 0.04 | -0.03 – 0.11 | 0.259 | 0.04 | -0.03 – 0.11 | 0.306 |
| **Carotid artery side (right) ^a^** |  |  |  |  |  |  | -0.02 | -0.04 – -0.00 | 0.045 |
| **New statin therapy (yes) ^a^** |  |  |  |  |  |  | -0.01 | -0.08 – 0.05 | 0.678 |
| **Smoking (yes) ^a^** |  |  |  |  |  |  | 0.02 | -0.07 – 0.10 | 0.713 |
| **Diabetes (yes) ^a^** |  |  |  |  |  |  | 0.04 | -0.04 – 0.11 | 0.380 |
| **Hyperlipidemia (yes) ^a^** |  |  |  |  |  |  | 0.08 | 0.00 – 0.15 | 0.037 |
| **Marginal R2 / Conditional R2** | 0.521 / 0.582 | | | 0.539 / 0.590 | | | 0.541 / 0.591 | | |

**Table VI:** Stepwise autoregressive linear mixed models for hemodynamic variables with wall thickness at follow-up as the dependent variable in the whole study cohort (=group 1).

^a^ Reference categories: female (sex), left (side), no (new statin therapy, smoking, diabetes, hyperlipidemia)

|  | Model 1 | | | Model 2 | | | Model 3 | | |
| --- | --- | --- | --- | --- | --- | --- | --- | --- | --- |
| **Predictors** | Estimates | CI | p | Estimates | CI | p | Estimates | CI | p |
| **(Intercept)** | 0.49 | 0.44 – 0.54 | <0.001 | -0.06 | -0.35 – 0.23 | 0.684 | 0.00 | -0.31 – 0.32 | 0.988 |
| **Baseline wall thickness** | 0.59 | 0.57 – 0.61 | <0.001 | 0.59 | 0.57 – 0.61 | <0.001 | 0.59 | 0.57 – 0.61 | <0.001 |
| **Oscillatory shear index (OSI)** | 0.05 | -0.04 – 0.15 | 0.283 | 0.05 | -0.05 – 0.14 | 0.324 | 0.03 | -0.06 – 0.13 | 0.478 |
| **Wall shear stress (WSS)** | -0.02 | -0.04 – -0.00 | 0.042 | -0.02 | -0.04 – -0.00 | 0.039 | -0.03 | -0.05 – -0.01 | 0.010 |
| **Age** |  |  |  | 0.01 | 0.00 – 0.01 | <0.001 | 0.01 | 0.00 – 0.01 | 0.003 |
| **Sex (male)^a^** |  |  |  | 0.06 | -0.01 – 0.13 | 0.106 | 0.06 | -0.02 – 0.13 | 0.137 |
| **Carotid artery side (right) ^a^** |  |  |  |  |  |  | -0.06 | -0.07 – -0.04 | <0.001 |
| **New statin therapy (yes) ^a^** |  |  |  |  |  |  | 0.06 | -0.02 – 0.13 | 0.129 |
| **Smoking (yes) ^a^** |  |  |  |  |  |  | -0.02 | -0.10 – 0.07 | 0.712 |
| **Diabetes (yes) ^a^** |  |  |  |  |  |  | 0.02 | -0.08 – 0.11 | 0.728 |
| **Hyperlipidemia (yes) ^a^** |  |  |  |  |  |  | 0.03 | -0.04 – 0.11 | 0.420 |
| **Marginal R2 / Conditional R2** | 0.370 / 0.464 | | | 0.401 / 0.476 | | | 0.405 / 0.479 | | |

**Table VII:** Stepwise autoregressive linear mixed models for hemodynamic variables with wall thickness at follow-up as the dependent variable in patients without ICA stenosis ≥10 % (=group 2).

^a^ Reference categories: female (sex), left (side), no (new statin therapy, smoking, diabetes, hyperlipidemia)


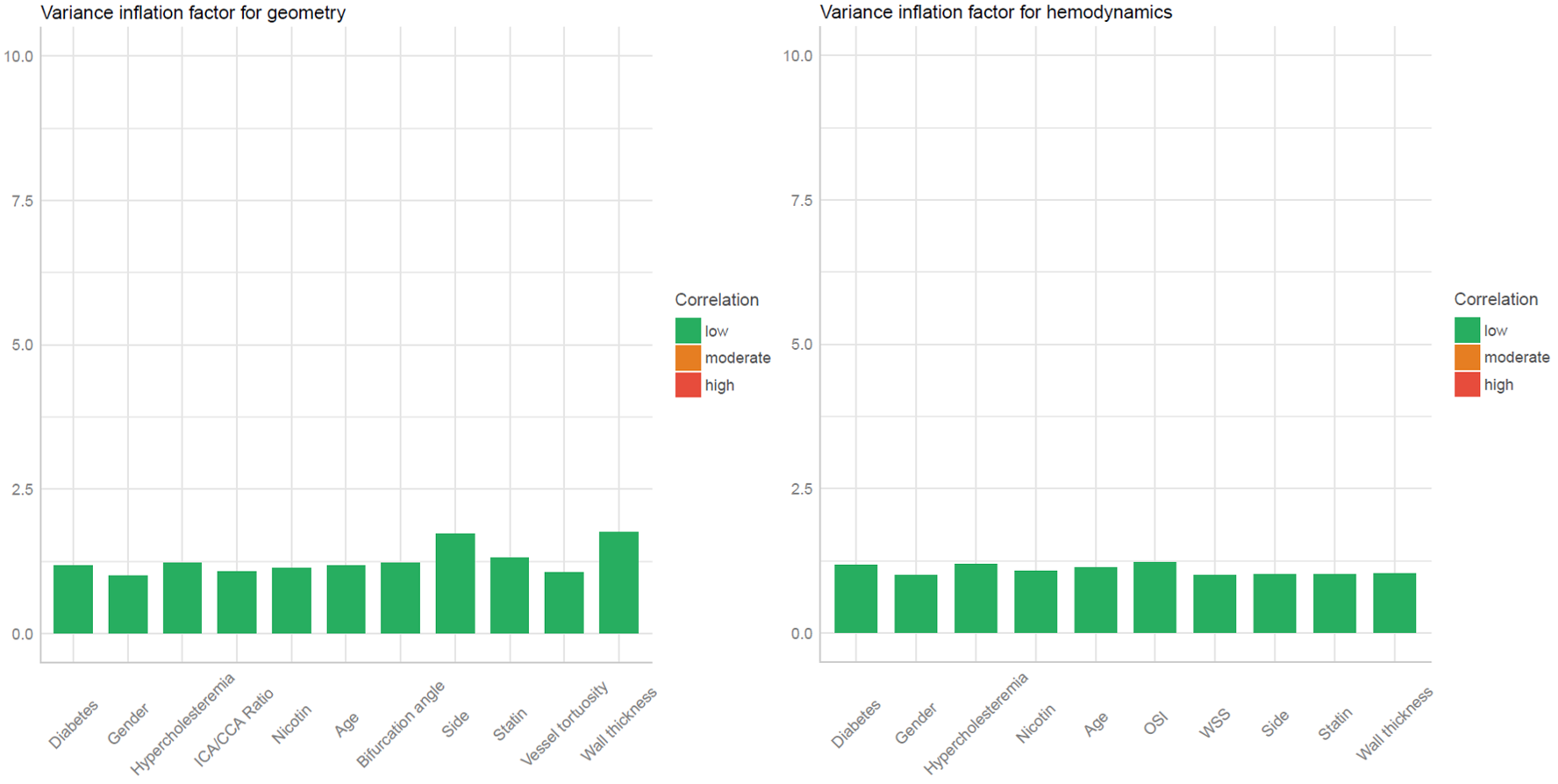


**Figure I:** Variance inflation factors for geometry (left) and hemodynamic factors (right).


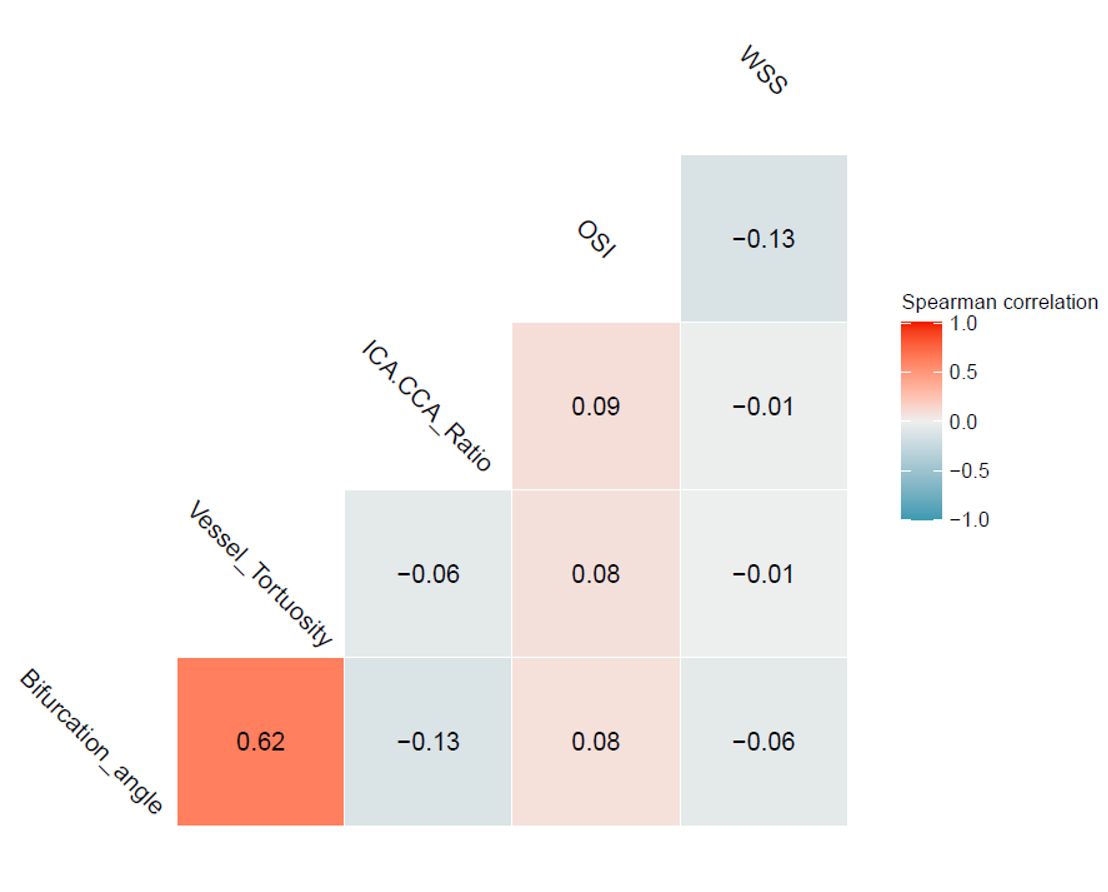


**Figure II:** Spearman correlations between geometry and hemodynamics
